# Road traffic accidents in Kathmandu—an hour of education yields a glimmer of hope

**DOI:** 10.1186/1757-7241-21-19

**Published:** 2013-03-21

**Authors:** Bibhusan Basnet, Rais Vohra, Amit Bhandari, Subash Pandey

**Affiliations:** 1 B.P. Koirala Institute of Health Sciences, Dharan, Nepal; 2 UCSF Fresno Medical Center, Fresno, CA, USA; 3 Nepal Emergecny Medicine Organization, Fresno, CA, USA

**Keywords:** Road traffic accidents, No drinking and driving policy, Behavioral program

## Abstract

After the Metropolitan Traffic Police, Kathmandu initiated a “No Drinking and Driving” policy in 2011 in which a major intervention for intoxicated drivers was mandatory 1-hour class to modify drunk driving behaviors, reports show that the number of road traffic accidents in the year 2012 decreased by 23 percent from the year 2011. The injury to fatality ratio decreased by 21 percent in this period. We remain encouraged by these statistics which confirm that increased enforcement of road traffic rules, combined with behavioral change programs, can have positive changes in LMICs which suffer considerably from the global burden of trauma.

## Correspondence

Respected Editor,

The article by Sakran et al. [1] published in your journal titled “Care of the injured worldwide: trauma still the neglected disease of modern society” referred to the fact that a number of Lower and Middle Income Countries (LMIC’s) have legislation in place for injury prevention (for example, seatbelt or helmet laws) but that a related problem lies in the adequate enforcement of these laws. Globally, Road traffic accidents (RTAs) remain the second highest cause of mortality in the adolescent and young adult age group, [1] but we have a success story to share from the traffic-dense capital city of Kathmandu which speaks to the importance of cooperation between law enforcement and public health agencies for the common good.

On Dec 3, 2011, the Metropolitan Traffic Police, Kathmandu initiated a “No Drinking and Driving” policy in which a major intervention for intoxicated drivers was a mandatory 1-hour class to modify drunk driving behaviors. The behavioral program included all the drivers who had minimal alcohol levels in the blood. It was based on structured, interactive sessions which covered: (a) driver education about the effects of increasing blood alcohol levels on the brain and motor control; (b) multimedia presentations demonstrating the harmful effects of driving under the influence of alcohol.

**Figure 1:**
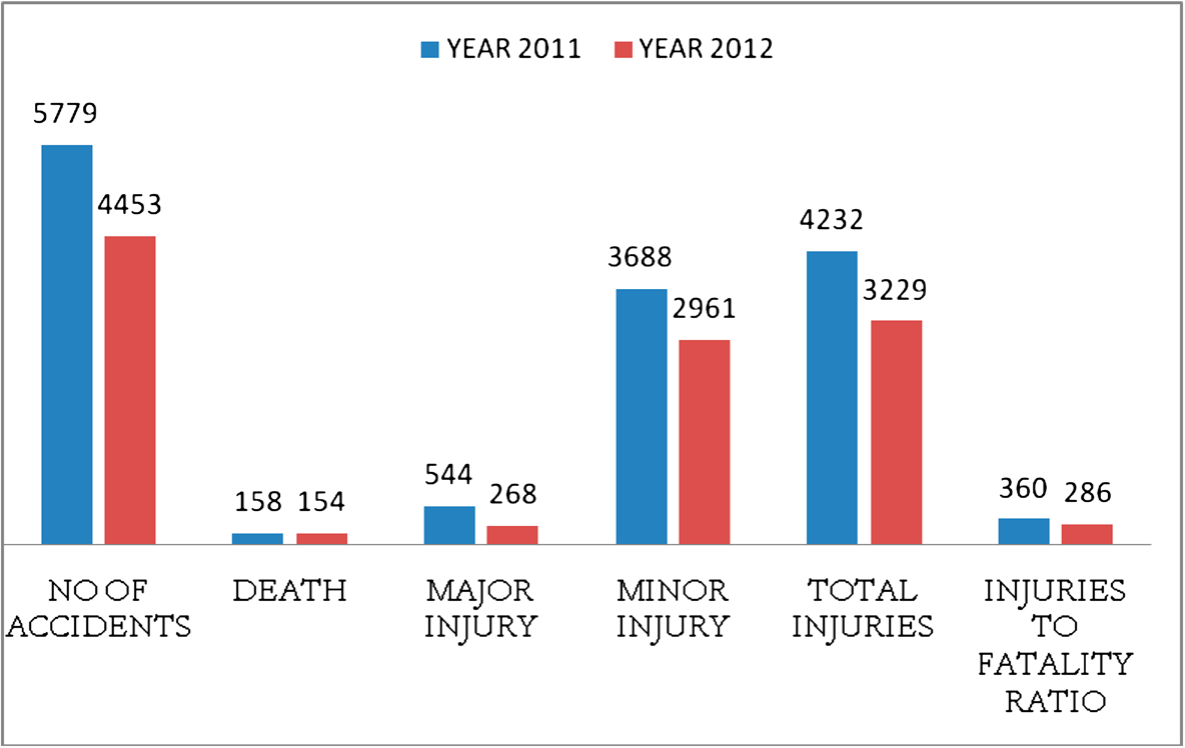
Road traffic accident outcomes in 2011 and 2012, one year before and after the intervention.

We analyzed data on RTAs and fatalities provided by the Metropolitan traffic police, Kathmandu for the 2 years straddling this intervention. Among the total injuries, 4232 major and minor road traffic accidents were seen in the year 2011, while 3229 accidents took place in the year when strict road traffic rules were followed/or implemented. Figure 1 shows the road traffic injuries and fatality rates during the pre- and post-intervention period.

The number of road traffic accidents in the year 2012 decreased by 23 percent from the year 2011. The injury to fatality ratio decreased 21 percent in this period. This positive change occurred even as there were increased numbers of vehicles on the already crowded roads of greater Kathmandu. Without a more robust trauma epidemiology or a citywide RTA registry, it is difficult to ascertain the role of the behavioral classes and increased police vigilance about drunk driving among other factors influencing driver behavior and clinical outcomes in central Nepal.

However, we remain encouraged by these statistics which confirm that increased enforcement of road traffic rules, combined with behavioral change programs, can indeed effect positive changes in LMICs which suffer considerably from the global burden of trauma.

## Competing interests

The authors’ declare that they have no competing interests.

## Authors’ contributions

BB, RV, AB, SP conceived and designed the study. BB, AB and SP collected the data. RV and BB wrote the first manuscript. All authors contributed, read and approved the final manuscript.

## Authors’ information

RV and BB are involved with Nepal Emergency Medicine Organization (NEMO) established since 2012, which is a non –profit organization based in Fresno, CA, USA and works on developing emergency medicine in Nepal.
